# Ketorolac versus Magnesium Sulfate in Migraine Headache Pain Management; a Preliminary Study

**Published:** 2017-01-08

**Authors:** Hossein Delavar Kasmaei, Marzieh Amiri, Ahmed Negida, Samaneh Hajimollarabi, Nastaransadat Mahdavi

**Affiliations:** 1 Department of Neurology, Shohadaye Tajrish Hospital, Shahid Beheshti University of Medical Sciences, Tehran, Iran.; 2 Department of Emergency Medicine, Shahid Beheshti Hospital, Guilan University of Medical Sciences, Anzali, Iran.; 3 Faculty of Medicine, Zagazig University, Zagazig, Egypt.; 4 Faculty of Medicine, Shahid Beheshti University of Medical Sciences, Tehran, Iran.; 5 Department of Anesthesiology, Torfeh Hospital, Shahid Beheshti University of Medical Sciences, Tehran, Iran.

**Keywords:** Magnesium sulfate, ketorolac, migraine disorders, emergency department, pain management

## Abstract

**Introduction:**

Migraine is a common cause of emergency department (ED) visits. To date, there is no recommended drug of choice for pain management of these patients. In the present study, we aimed to evaluate the effectiveness of ketorolac and magnesium sulfate in this regard.

**Methods:**

This is a cross-sectional study performed on all 18 - 60 year-old patients, visiting two different EDs with complaint of moderate to severe migraine headache. Patients were treated with 30 mg ketorolac in one hospital and 1 gram magnesium sulfate in the other. Pain scores were assessed on arrival, 1 and 2 hours after drugs administration and quality of pain management was compared between two groups using SPSS 22.

**Results:**

70 patients with the mean age of 36.4 ± 11.4 years were enrolled (51.4% male). The two groups were similar regarding baseline characteristics (p > 0.05). The improvement in pain score in magnesium sulfate group was greater than Ketorolac group after both one hour (6 vs 3; p < 0.001) and two hours (7 vs 5; p < 0.001).

**Conclusion:**

It seems that both ketorolac and magnesium sulfate are significantly effective in pain control of patients with migraine headache presenting to the emergency department. Magnesium sulfate was superior to ketorolac both one and two hours after drug administration.

## Introduction

Migraine is a disabling disorder and a common cause of emergency department (ED) visits (1, 2). Migraine prevalence has been reported to be 12-20% in different populations (3). To date, various drugs such as opium, nonsteroidal anti-inflammatory drugs (NSAIDs), neuroleptics, and triptans have been used for treatment of migraine headaches in ED (4-7). Numerous studies have been carried out to evaluate the effectiveness of above-mentioned drugs, but to date there is no recommended drug of choice for this purpose. Magnesium sulfate and NSAIDs such as ketorolac are among the common drugs used for migraine headache pain management (8-14). 

In the present study, we aimed to evaluate the effectiveness of ketorolac and magnesium sulfate in controlling migraine headaches in patients presented to ED.

## Materials and methods

***Study design and setting***

This is a cross-sectional study performed from April to October 2015 in emergency departments of Imam Hossein and Shohadaye Tajrish Hospitals, Tehran, Iran, to compare the effectiveness of ketorolac and magnesium sulfate in migraine pain management. 

***Ethical issues***

The protocol of the study was approved by the Ethical Committee of Shahid Beheshti University of Medical Sciences. The authors declare their adherence to ethical principles of Helsinki Declaration throughout this research. Eligible patients were enrolled after signing informed consent form. 

***Participant***

All 18 - 60 year-old patients, visiting the emergency department with complaint of moderate to severe headache were interviewed by the main investigators. They were included if compatible with the international headache society (IHS) criteria for common migraine and had a pain score more than 5 (based on visual analogue scale (VAS)). Patients with history of peptic ulcer disease, ischemic heart disease, inflammatory bowel disease, coagulopathy, renal or hepatic failure, history of recent major surgery, history of hypersensitivity to the studied drug, and hypertension, as well as pregnant or breastfeeding women were excluded.

***Studied groups***

Patients in Shohadaye Tajrish Hospital were treated with 30 milligrams ketorolac and those in Imam Hossein Hospital with 1 gram magnesium sulfate. One of the investigators in each hospital assessed pain scores on arrival, 1 and 2 hours after drug administration, when plasma concentration reaches its peak level. Patients, pain investigator, and data analyzer were blind to the administered treatment. 3 or more scores decrease in VAS was considered as clinically significant pain management.

***Statistical analysis***

Statistical analysis was performed using SPSS version 22. Continuous variables were described as mean ± standard deviation, and categorical ones as median, interquartile range, frequency and percentage. Mann-Whitney U test and Wilcoxon test were used to analyze differences in VAS pain scores. P value < 0.05 was considered significant. 

**Table 1:** Baseline characteristic of enrolled patients

**Variable**	**Ketorolac**	**Magnesium sulfate**	**P**
Age; mean ± SD	36.9 ± 10.7	36.0 ± 12.5	0.73
Gender; male number (%)	15 (42.9)	19 (54.3%)	0.33
Analgesic consumption before arrival; number (%)	18 (51.4)	12 (34.3)	0.14
Duration of analgesic consumption before arrival; mean ± SD (hour)	3.0 ± 2.08	3.4 ± 1.6	0.56
Pain score on arrival; median (IQR)	8 (3)	8 (2)	0.15
Pain score after 1 hour; median (IQR)	4 (4)	2 (2)	<0.001
Pain score after 2 hours; median (IQR)	3 (2)	0 (1)	<0.001

SD: standard deviation, IQR: inter quartile range.

**Figure 1:**
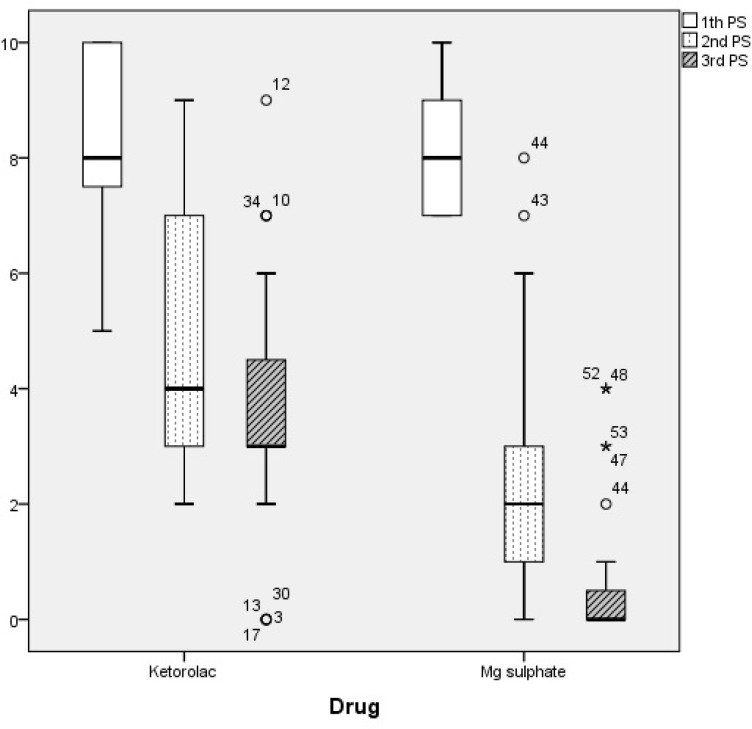
Box plot comparing minimum, maximum, median and inter-quartile range of pain scores at baseline, 1 hour, and 2 hours after drug administration between ketorolac and magnesium sulfate groups (p < 0.001

## Results

Seventy patients with the mean age of 36.4 ± 11.4 years were enrolled (51.4% male). Baseline characteristics of patients in each group, as well as their VAS scores at baseline, 1 hour, and 2 hours after drug administration are compared in table and figure 1. The improvement in VAS pain score in magnesium sulfate group was greater than Ketorolac group after both one hour (6 vs 3; p < 0.001) and two hours (7 vs 5; p < 0.001). 

## Discussion

Based on data extracted from the current study, both ketorolac and magnesium sulfate are significantly effective in pain control of patients with migraine headache presenting to the ED. Magnesium sulfate was superior to ketorolac one and also two hours after drug administration. 

These drugs have been studied in patients with migraine headache solely or in comparison with other drugs. However, searching in Google Scholar and Pubmed database with “ketorolac and magnesium sulfate and migraine” as keywords, did not find any article that compared these two with each other. The findings of the literature have yielded conflicting results. A paper review performed in 2000 on the effectiveness and safety of different therapies in migraine headache, revealed that magnesium sulfate needs to be studied in appropriate trials before a conclusions can be drawn. Ketorolac performs a little better but has been shown to be inferior to other treatments (15). 15 years later in 2015, based on published literatures between 1998 and 2013, American Headache Society reported that both ketorolac and magnesium sulfate are probably effective for acute migraine treatment (Level B evidence)(16). Simultaneously, Canadian Headache Society strongly recommended the use of ketorolac, based on a low level of evidence and weakly recommended against the use of magnesium sulfate, based on moderate-quality evidence (17). High quality papers that have been published recently in authentic journals, clarify the ambiguity surrounding this topic. The current study still cannot prove anything with high reliability; but can be considered as a pilot study for running further appropriate double blind randomized controlled trials. 

## Conclusion:

It seems that both ketorolac and magnesium sulfate are significantly effective in pain control of patients with migraine headache presenting to the emergency department. Magnesium sulfate was superior to ketorolac both one and two hours after drug administration. 
